# Community-Based Portable Reefs to Promote Mangrove Vegetation Growth: Bridging between Ecological and Engineering Principles

**DOI:** 10.3390/ijerph18020590

**Published:** 2021-01-12

**Authors:** Sindhu Sreeranga, Hiroshi Takagi, Rikuo Shirai

**Affiliations:** Department of Transdisciplinary Science and Engineering, School of Environment and Society, Tokyo Institute of Technology, Meguro, Tokyo 152-8550, Japan; sindhu.s.aa@m.titech.ac.jp (S.S.); shirai.r.aa@m.titech.ac.jp (R.S.)

**Keywords:** young mangroves, mangrove restoration, portable reef design, field observation, Amami Oshima

## Abstract

Despite all efforts and massive investments, the restoration of mangroves has not always been successful. One critical reason for this failure is the vulnerability of young mangroves, which cannot grow because of hydrodynamic disturbances in the shallow coastal water. For a comprehensive study bridging ecological and engineering principles, a portable community-based reef is proposed to shield mangroves from waves during the early stages of their growth. A series of field observations were conducted on Amami Oshima Island (Japan), to observe the growth of young mangroves and their survival rate under moderate wave conditions. The evolution of young mangroves was also observed in the laboratory under a controlled indoor environment. At the research site, it was confirmed that, after six months of germination, young mangroves could withstand normal high waves. Laboratory-grown plants were lower in height and had fewer leaves compared with the native mangroves on Amami. Based on these results, an economical reef system was designed. For this purpose, the Ahrens formula for the design of a low-crested reef breakwater was revisited. The results showed that a 50-cm-high reef constructed with 15-kg stones can protect mangroves that are a few months old and effectively promote early mangrove growth.

## 1. Introduction

Mangrove forests are the most productive ecosystems on the planet among various marine ecosystems [1]. The leaves and roots that filter the salt from seawater enable mangroves to survive in the high tide, while they absorb oxygen for photosynthesis during low tide [2]. Mangroves play a crucial role in protecting coastal regions by reducing the damage caused by tsunamis, storm surges, and tropical cyclones and in saving human settlements. Several studies on the 2004 Indian Ocean tsunami reported that the loss of human life was significantly lower in the presence of mangrove forests, although it was also dependent on the distance and elevation of human settlements from the coastline [3,4,5]. An interview-based survey revealed that local people in the Philippines believed that mangroves protected their lives from the historical event of Typhoon Haiyan in 2013 [6].

The dynamic interaction between the mangrove system and ocean waves is not fully understood, it is believed that mangroves reduce wave energy and promote sedimentation [7,8,9]. Mangroves work as a barrier, leading to changes in flow direction resulting in vegetative surface friction, inducing wave energy dissipation and damping [10]. The advantages of mangrove forests are referred to as “ecological resilience” for their ability to absorb hydrodynamic disturbance [11]. Mangroves are also considered to be a green infrastructure that contributes to disaster prevention through flood regulation, erosion control, sediment trapping, nutrient recycling, wildlife habitat, and nurseries [1,12,13,14,15,16].

The mangrove area has been reduced by 45% in the past 23 years, shrinking its geographical coverage from 137,760 km^2^ [17] to 81,484 km^2^ [18] worldwide. The declining rate is most significant in developing countries in Asia [19,20]. If such degradation continues, mangroves may disappear in the next 100 years [21]. This degradation is triggered by multiple factors, such as urban development, industrialization, agricultural land expansion, timber and charcoal production, and shrimp farming [16,20,22,23]. In Indonesia, Thailand, and Malaysia, the loss of mangrove forests is triggered by the conversion of land to aquaculture, agriculture, and salt production [23,24]. Tin mining and wood harvesting are also major causes of mangrove degradation in Thailand [24]. Half of the cleared mangroves in Southeast Asia and in South and Central America were due to fish and shrimp aquaculture [25,26]. Conservation actions have been implemented globally to compensate for the loss from deforestation, aquaculture, urban development, industrialization, and shrimp farming. Large-scale mangrove plantations were created in many countries of South and Southeast Asia by nongovernmental organizations and nonprofit organizations such as Wetland International (WI) and the International Union for the Conservation of Nature (IUCN) [22,27]. The Mangroves for the Future initiative was set up through the collaboration of multiple international agencies, such as the United Nations Development Programme, United Nations Food and Agriculture Organisation, and IUCN, to promote the sustainable conservation of coastal ecosystems [27]. The collective efforts of these programs have emphasized the sustainability of coastal ecosystems. The cost of mangrove restoration projects has varied from 1 to 10 million USD, as observed in projects that have taken place in Pakistan, Indonesia, Vietnam, the Philippines, and Senegal [27].

In addition to these global initiatives, scientific communities are trying to develop new ideas for protecting coastal areas from coastal hazards by incorporating the mangrove ecosystem for ecological disaster risk reduction (Eco-DRR). Eco-DRR is an effort composed of the restoration, conservation, and sustainable management of ecosystems to reduce the risk of disaster [11]. The idea of rehabilitating mangroves on a hybrid raised platform proposed recently [12] is expected to lead to new strategies for disaster risk reduction. Ideally, mangrove replantation and conservation should be implemented as a community-based approach (CBA) to improve the preparedness of the local community in response to coastal disasters [28]. However, it has been reported that the success rate of large-scale restoration is not necessarily high [27,29], creating a difficult situation for further dissemination of the mangrove rehabilitation program globally [30,31]. A success rate of only 10–20% was achieved in a community-based restoration program in the Philippines because of inappropriate species and site selection [30,32], while 40% of mangrove seedlings vanished and a 60% success rate was achieved in a similar attempt in Sri Lanka [33]. The principal reasons for these failures are thought to be physical factors (e.g., unusually high waves and less sediment supply [30]) and biological factors, such as the death of seedlings resulting from the dense growth of algae, sapling damage by insects, eating away of young seedlings by aramid crabs, and increase in predation rates by crabs on mangrove plants [31]. Biochemical factors, such as deficiencies of carbon, nitrogen, phosphorus, and other organic matter in sediments, can lead to failure to maintain healthy mangrove seedlings [34]. It is difficult to measure the success and failure of mangrove rehabilitation efforts because of inadequate documentation, particularly when a project fails [32].

Hydrological factors, such as tides, wind-generated waves, and currents, significantly influence the growth phase of young mangrove plants. Wave actions are higher in the wetland rehabilitation sites, causing flooding and damaging young mangrove seedlings [31]. Waves uproot the seedlings, mainly where propagules did not root firmly on loosely deposited sediments [32,35,36,37]. In Colombia, 93% of seeds died during the initial four months during a prolonged period of flooding. However, the high mortality ratio was not necessarily caused by the inundation, but the uprooting of seedlings in soft sediments as a result of wave actions was also responsible [35].

Simple countermeasures have been implemented to protect mangrove conservation areas by local communities, e.g., constructing barriers made of rocks, logs, and sand bars to attenuate wave actions and trap sediments [31]. Portable and inexpensive materials are also preferable because they can be used for construction by local communities. Wooden piles were also tested in Thailand and Vietnam; however, the effectiveness of these methods has not been sufficiently proved [38]. As a negative effect, they may even cause erosion and sediment destabilization, affecting the natural mangrove settlements [31,39]. Ecological engineering perspectives and applications are considered important steps in restoration [40]. However, the National Research Council of United States, stated that there are barriers to implementing coastal engineering principles in mangrove restoration projects because they are usually costly [40,41]. WI’s Mangrove Action Project identified the failed planting techniques and emphasized the necessity of a new approach based on lessons learned from the failed projects [42].

**Figure 1:**
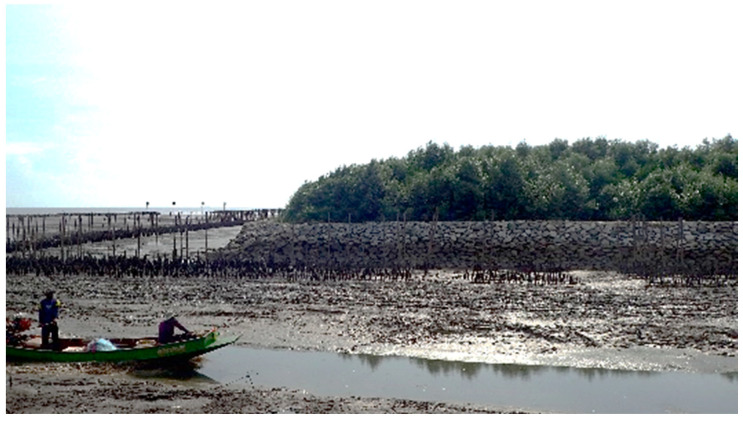
Successful mangrove plantation in Chonburi, Thailand—however, the rubble dike stopped the expansion of the forest [photo taken by one of the authors].

As previously reported, mangrove reforestation did not adequately incorporate engineering principles. The aim of present study was to bridge the ecological and engineering approaches. For example, a stone dike was used for mangrove reforestation (Figure 1). However, in the end, the expansion of the mangrove forest was stopped owing to the presence of the dike. Hence, the size of the stone should be carefully designed so that the dike can be demolished at a later stage. In this study, a CBA called a “portable reef” was developed to protect mangrove plants from hydraulic disturbances. The reef was designed to achieve low-cost coastal protection by placing portable rubble or blocks in front of mangrove plantation areas. It may take several months for mangroves to grow sufficiently to withstand high waves. Therefore, a barrier must sustain its function, at least during the first several months after plantation [12]. Accordingly, a portable reef for only the very early stage of the plantation would have a simple structure. Once mangrove plants grow sufficiently, the portable reef can be dismantled and relocated to other locations for another community activity use. To confirm the feasibility of this concept, the present study has two parts: (i) field and laboratory observations were conducted to understand the basic ecology and growth rate of young mangroves, and (ii) the minimal design requirement for an efficient portable reef was identified.

## 2. Materials and Methods

Mangrove growth was observed in the field and the laboratory for approximately six months. The findings were used in designing a portable reef with an emphasis on reducing stone weight, which is essential for community-based construction.

### 2.1. Field Survey

**Figure 2:**
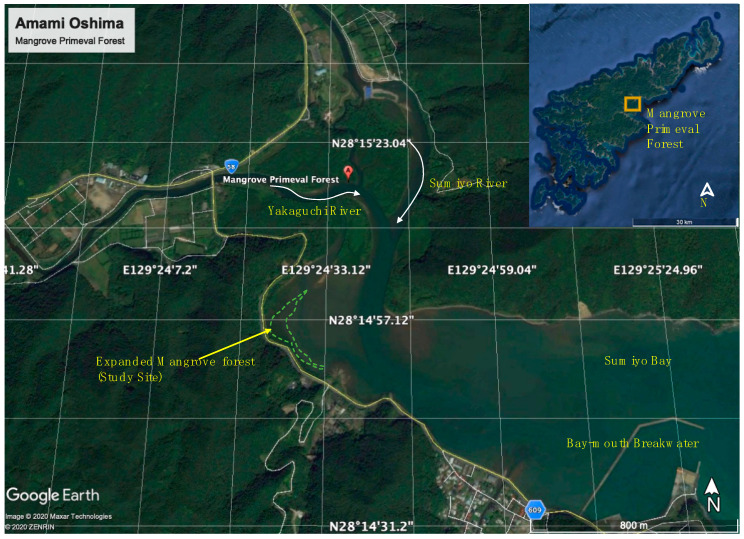
Mangrove study site, which is located at the inner-most part of Sumiyo Bay in Amami (study site location: 28°14′57.3″ N 129°24′24.8″ E).

Field surveys were conducted in May 2019 and December 2019, and seaward expansion of young mangrove shrubs was found on sediment deposition in the tidal inlets of Amami Oshima Island (hereinafter “Amami”), Japan. The month of May is the postpollination period, in which the species *Kandelia obovata* in the primeval mangrove forest produces enormous seeds, whereas December is cold, and mangrove growth is not vibrant [43]. A large colony of natural mangroves (*K. obovata*) was identified in Sumiyo Bay of Amami near the inlets of the Yakugachi River and Sumiyo River (Figure 2). As observed in the field survey, mangrove seedlings were transported from the mainstream of the primeval forest and settled on the shallow mudflat with the fast-developing root system. While many mangrove forests are facing degradation in Japan, the primeval mangrove forest of Amami has been expanding for several decades [44]. This mangrove forest is located in areas where the flow of rivers is mild, and the coast is shallow and calm. When the tide recedes, the tidal flat becomes a place where organic matter from the river and the ocean is deposited, providing a habitat for a variety of animals. In addition to the favorable environmental conditions, the expansion of the area may also partially be attributed to the breakwater constructed at the bay mouth, protecting the inner bay area from offshore high waves (Figure 2).

**Figure 3:**
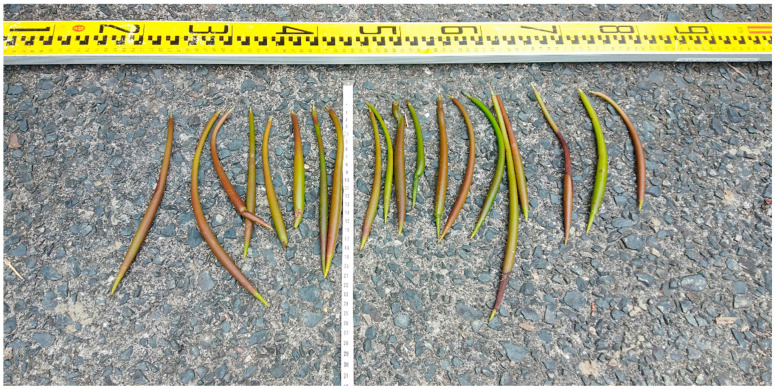
Mangrove seeds (propagules of *K. obovata*) collected in the Amami study site.

**Figure 4:**
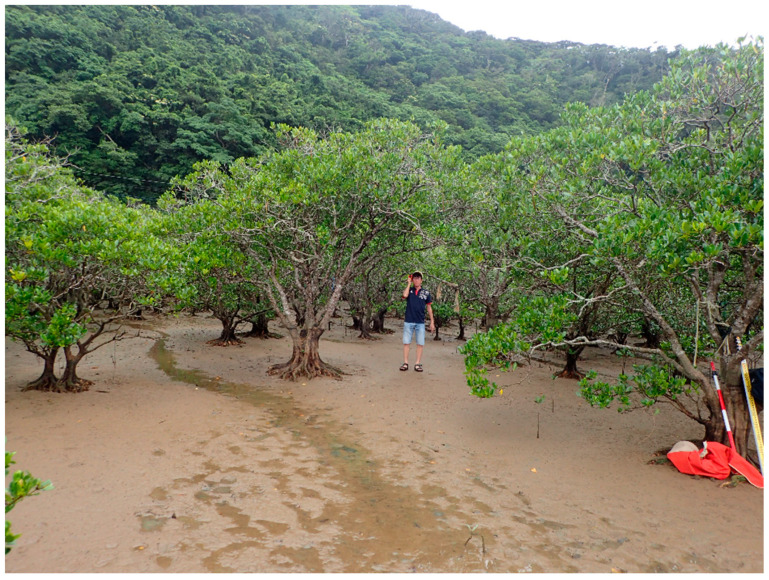
Measurement of the topography in the mangrove forest.

During the survey conducted in May, 40 mangrove propagules were collected to measure the size (Figure 3). Half were used for the plantation test in the Amami primeval mangrove forest, and the rest were transported to a laboratory in Tokyo. A topography survey was conducted to measure the ground level within the mangrove zone. The salinity level and water temperature were recorded. An aerial survey was conducted using a drone (Phantom 4 Pro; DJI Technology Co. Ltd., Shenzhen, China) to observe mangrove shrub density in near-shore, midshore, and offshore regions. Drone images were validated with field observations to confirm the densities of the mangrove plants. The elevation of the mudflat was measured using laser range finders (TruPulse 360; Laser Technology Inc., Centennial, CO, USA) (Figure 4). The predominant mangrove species observed in the study site was *K. obovata*, which is a dwarf-type tree often found in India, Singapore, Cambodia, Malaysia, the Philippines, Indonesia, Myanmar, Bangladesh, Thailand, and Vietnam [45]. The genetic and phenotypic segregation suggests that the species *K. candelin* originated from some parts of China and Japan, and it is now classified as a new species, *K. obovata* [46]. This species is often found in the intertidal region of an estuary, which is frequently inundated by tides, like other Rhizophora mangrove species [40]. *K. obovata* in the Amami region produces seeds, particularly between the months of May and August, which are suspended by tidal flow to colonize themselves in new locations.

During the December 2019 survey, the number of surviving mangrove plants, plant height, number of leaves, root length, stem thickness, and stem color were investigated. The plant age was estimated based on the growth rate between the two surveys. These parameters were then compared with those of laboratory-grown plants under a controlled environment. An in situ manual wave-generating test using a paper board was also conducted to test the failure limitation of young mangrove plants against waves. Additionally, the salinity level, water temperature, turbidity, current velocity (FP111; YSI Inc., Yellow Springs, OH, USA), and water depth at high tides were measured during this second campaign. Water levels were recorded using pressure gauges for approximately 2 h (DEFI2-D10; JFE Co. Ltd., Chiba, Japan). The measured parameters were further considered in the conceptual design of the portable reef.

### 2.2. Laboratory Test

Twenty seeds of *K. obovata* were transported to the laboratory in Tokyo and planted in a range of different types of soil: (i) Amami’s native soil, (ii) a mixture of sand, silt, and compost, and (iii) coastal sand taken in Tokyo. The growth of these plants was monitored for approximately six months from June to December 2019. The duration of laboratory observations was consistent with that of the in situ plantation test in Amami. The laboratory growing test was conducted to monitor the growth of mangroves in a controlled environment. The test simulated a situation where mangrove plantations are started from seedlings that were originally grown in a pot. Another important idea behind the field survey and laboratory plantation was to investigate the early growing stage of mangrove plants, because the design of the portable reef depends highly on initial plant growth.

## 3. Field Observation Results

### 3.1. Survey in the Study Site

**Figure 5:**
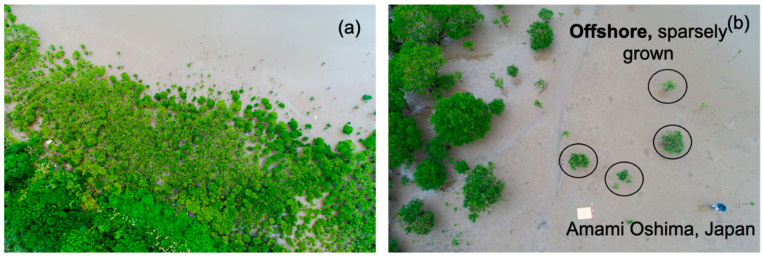
(**a**,**b**) Observed mangrove density at the study site in Amami (photos were taken by a drone).

The land slope of a mangrove forest approximately 80 m wide was measured to be as mild as 1/100 on average. This can be considered as a gentle slope but not extremely flat. The density of mangrove plants varied extensively depending on location, i.e., offshore, midshore, and near-shore regions. When approaching the offshore area, it was observed that the number of plants tended to decrease. The lowest mangrove densities were found offshore, where water depths reached half a meter at high tide. Mangrove density was very sparse offshore, whereas 2–4 plants/m^2^ in the midshore and 10–20 small mangrove plants/m^2^ around two matured shrubs onshore were observed, as shown in Figure 5a,b.

### 3.2. Mangrove Plantation Test at the Research Site

**Figure 6:**
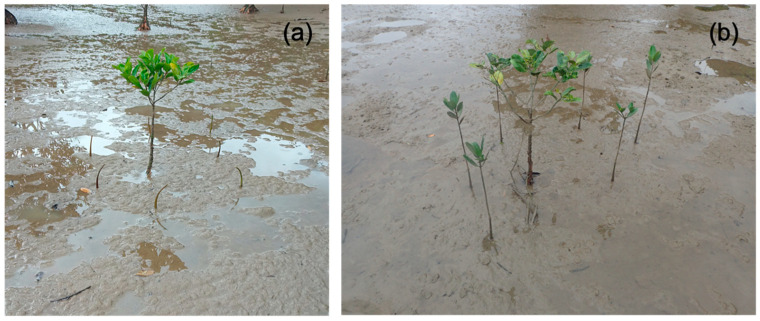
(**a**) Mangrove seeds planted around a prominent existing mangrove in May 2019 and (**b**) mangrove plant growth in December 2019.

**Figure 7:**
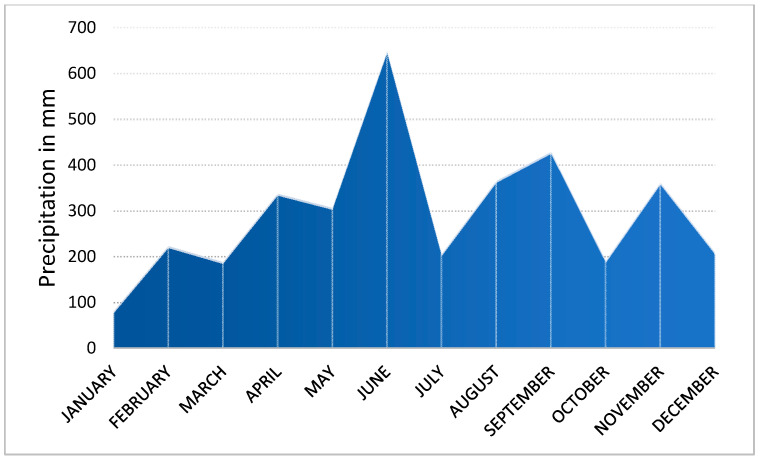
Monthly precipitation in 2019, recorded at Naze WMO Station in Amami (Lat 28°22.7′ N Lon 129°29.7′ E)—source: Japan Meteorological Agency.

Figure 6a,b illustrate the plantation of mangroves and their growth from May 2019 to December 2019. Twenty seeds with an initial seedling length of 18.5 cm on average (standard deviation of 3.5 cm) were planted in the offshore zone. The diameter of the seeds varied among the seedlings, with the widest part being 5–10 mm. As shown in Figure 3, some seedlings were completely straight, whereas the others were significantly curved, as observed during the plantation. The color of the seeds varied from green to brownish-green. Of the many plantations, one was made around an existing mangrove plant to clearly identify it during a future survey, as in Figure 6a,b, and the remaining seedlings were planted in nearby locations. In the December 2019 visit, it was found that the survival rate of planted seedlings was 75% (15 out of 20 seeds). Hydrological disturbances, such as high waves, unusual tides, and currents, especially during the typhoon season (July to October), can result in the uprooting of seedlings in soft sediments. Fortunately, however, no strong typhoon approached Amami during the half-year of this survey. Nevertheless, the five seedlings died or washed away for some reason. Various microbial organisms, such as bacteria, fungi, viruses, nematodes, and insects [47], and abiotic factors, such as high salinity levels and low and extremely high temperatures, could have adversely affected the growth of the mangrove plants [2]. The loss may also have been caused by sapling damage by animals, e.g., sea crabs, which selectively eat young seedlings. The water temperature and salinity level in May 2019 were 28.3 °C and 8‰, respectively, while the temperature reduced to 26.1 °C and the salinity increased to 19‰ in December 2019. The rise in salinity may have been because the water discharge from the two rivers is higher in spring than in winter. The precipitation chart is shown in Figure 7, which also supports the observation that salinity levels might have varied because of the difference in precipitation, which is higher in the rainy season (around June) and lower in the winter season (around December).

### 3.3. Water-Level Observations

**Figure 8:**
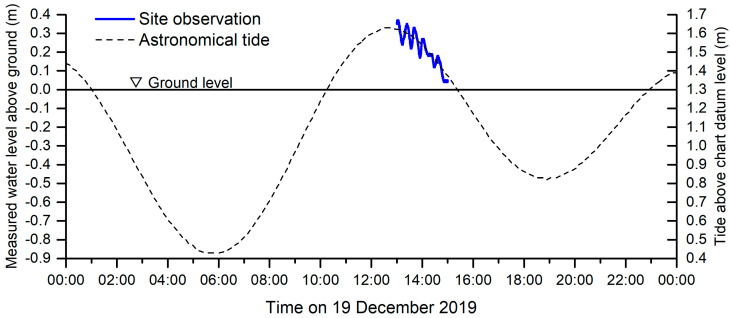
Water levels recorded at 1-s intervals for approximately 2 h (19 December 2019) presented in coordination with local astronomical tide data of the day.

Figure 8 depicts water levels and their fluctuations in the study site, recorded using the pressure sensor, on the one-day cycle of the predicted astronomical tide of the bay. The water level fluctuates in correspondence with astronomical tides. It dropped from nearly 0.4 to 0.05 m above the ground level in 2 h, between high to medium tidal ranges. This observation was conducted on a half-moon day during a medium tidal phase. However, the actual water level oscillated with a short period of approximately 20 min on top of the tidal curve. Although it has not been confirmed, this seems to be a sort of seiche that occurs in the bay. As a result, actual currents in mangrove forests may be faster than those induced by pure astronomical tidal forcing. However, the maximum velocity measured in the field was approximately 10 cm/s. This is significantly slower than the velocity generated in a tidal-dominant river mouth where a tidal current of more than 1 m/s often occurs [48]. Because the water depth is very shallow in the study site, frictional effects are believed to be responsible for a significant reduction in flow speed. This level of tidal currents is considered to be less impactful to young mangroves.

### 3.4. In Situ Wave Experiment

**Figure 9:**
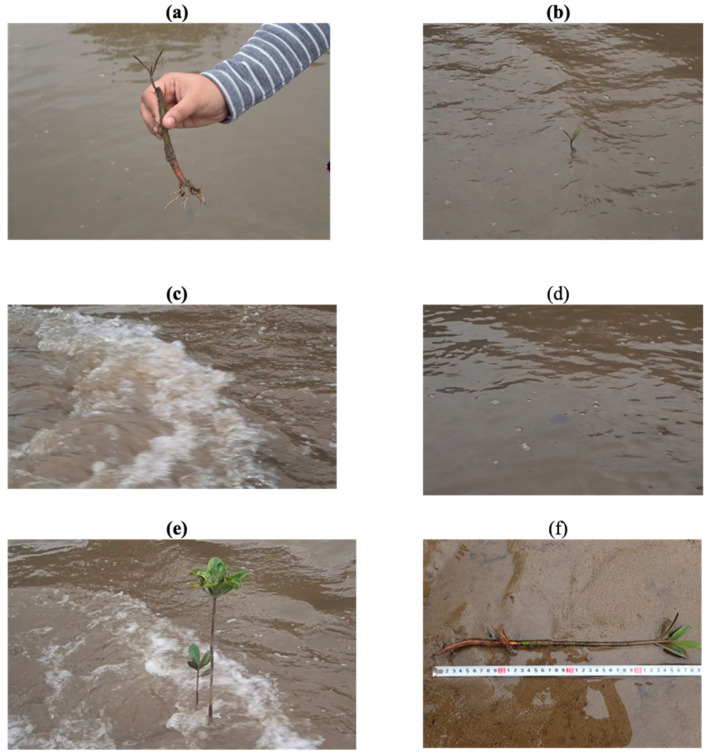
On-site manual wave generating test: (**a**) one-month mangrove, (**b**) before the wave test, (**c**) during the wave test, (**d**) after the wave test, (**e**) half-year-old and one-year-old mangroves during the wave test, and (**f**) half-year-old mangrove pulled out.

In the December survey, an in situ wave experiment was conducted to observe the strength of plants against waves. Waves were manually generated with a paper board for approximately 2 min, which impacted different age groups of plants, such as a month, a half year, and one year. The purpose of this test was to verify the differences in response to waves and critical wave height among the three young mangroves in different growth stages. The maximum wave height during manual wave generation was estimated to be approximately 10 cm through visual analysis with a video image. Figure 9a shows a 17-cm-long one-month-old mangrove that had two leaves, a short main root of approximately 2 cm, and thin subroots. The plant was mostly submerged during the test (Figure 9b). The one-month-old plant was broken entirely and submerged by a 2-min continuous-wave impact, as in Figure 9c,d, whereas a half-year-old and one-year-old mangrove 39 and 80 cm in height, respectively, survived without visible damage, as shown in Figure 9e. The six-month-old mangrove plant shown in Figure 9f developed a stiff root system with a length of one third the total plant length. Hence, it was firmly rooted in the sediment and could withstand the waves. The field investigation revealed that mangroves in the very early stage of growth (a few months) were particularly weak, whereas a half-year-old or older mangrove can sufficiently withstand moderate waves. Thus, special protection is required to protect mangrove seedlings from high waves in the initial two to three months. As a rough estimate, the failure limit of mangroves is considered to be a wave height of 0.1 m, which was used as a basis for the structural design of a portable reef.

### 3.5. Comparison of Mangrove Growth between Laboratory and Field Experiments

**Figure 10:**
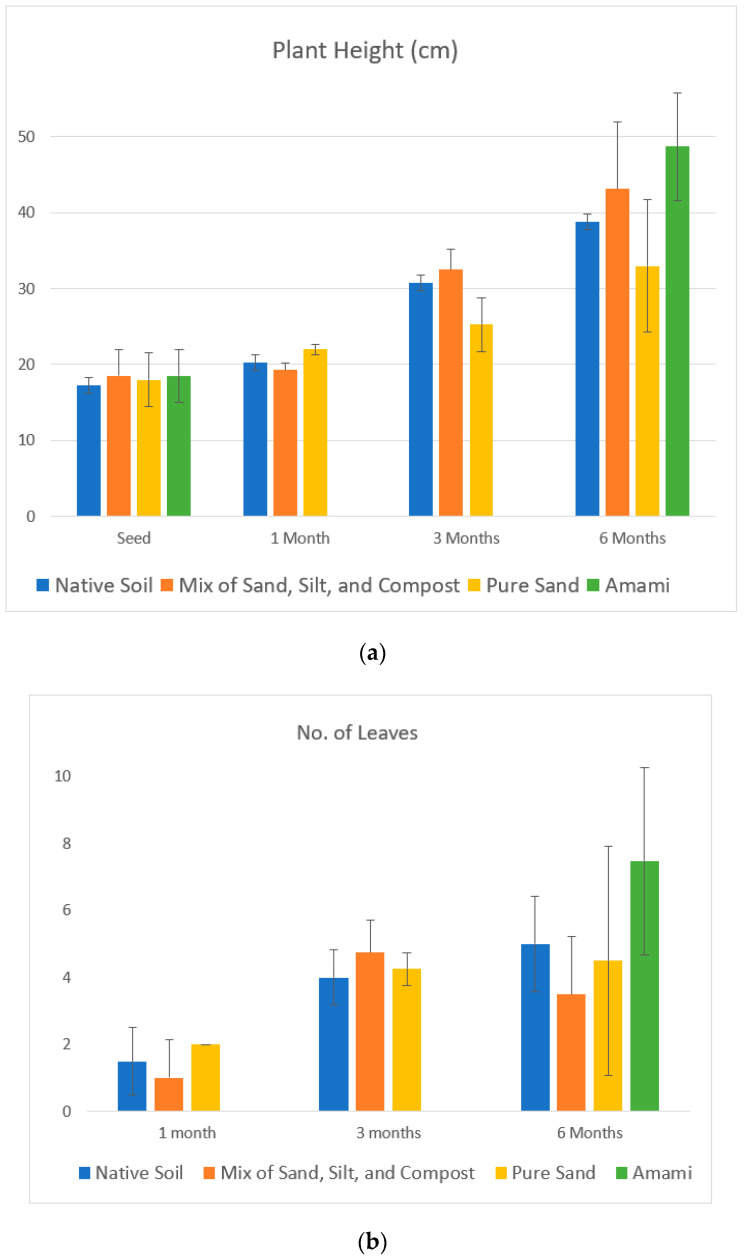
(**a**,**b**) Average mangrove growth in terms of height above the soil and the number of leaves recorded during six months in the laboratory: error bars indicate the range of minimum to maximum.

**Figure 11:**
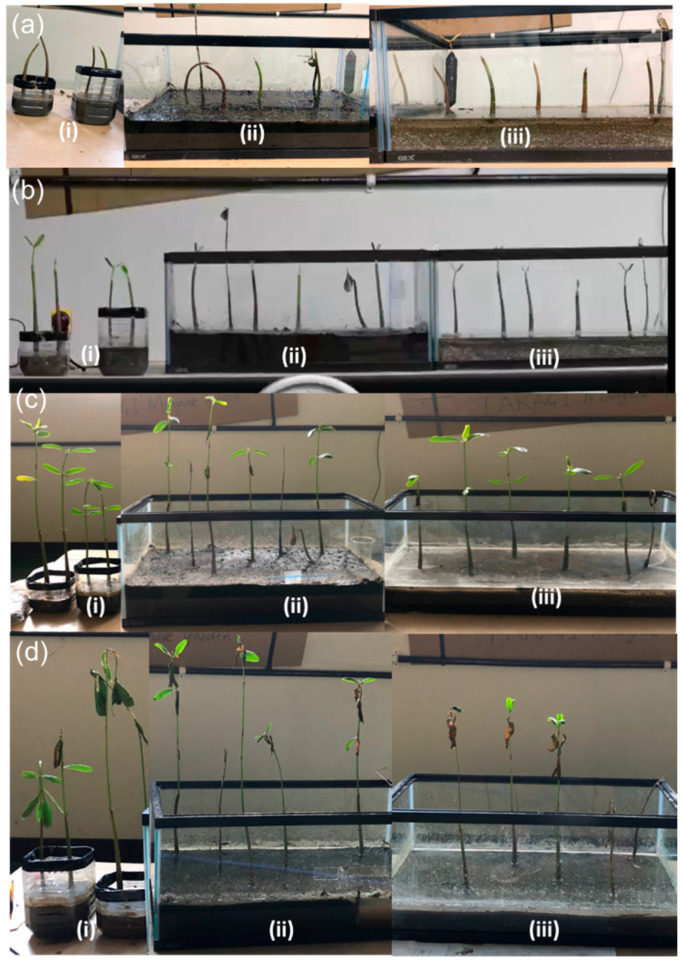
Growth of laboratory mangroves in (i) native soil, (ii) mix of sand, silt, and compost, and (iii) pure sand (**a**) on day of the plantation (5 June 2019), (**b**) after one month (10 July 2019), (**c**) after three months (9 September 2019), and (**d**) after sixth months (10 December 2019): an example of the growth record has been uploaded as a time-lapse video at http://www.ide.titech.ac.jp/~takagi/Labmangrove.html.

Figure 10a,b illustrate the mangrove evolution in terms of plant height and number of leaves in the laboratory. Mangrove growth was also measured six months after plantation at the Amami research site, as shown in Figure 6b, and compared with the sixth-month growth of plants in the laboratory. In the laboratory in Tokyo, the growth of the mangrove plant was observed for six months, and measurements were taken for the first, third, and sixth months. Pregerminated mangrove seeds (propagules) brought from the site were planted to observe their growth in different soil states: native soil, a mixture of sand, silt, and compost, and pure sand. The growth of mangroves was observed and measured in terms of the average height and the average number of leaves with standard deviations, as shown in Figure 10a,b. Pictures of propagule growth were taken at zero, one, three, and six months of laboratory plantation, as shown in Figure 11a–d. Although the initial growth was similar among the three soil types, the fastest plant growth was observed in the mixture of sand, silt, and compost after six months. Two plants in the mixture soil had withered at the end of the sixth month, despite showing good growth until three months. The average height of mangrove plants in Amami was 49 cm, with an average of 7.3 leaves per plant, as in Figure 10a,b, while indoor plants demonstrated growth of a height of 38.5 cm with the number of leaves up to 4.3 on average. The laboratory-grown plants were 21% lower in height than the plants grown in Amami. Similarly, the average number of leaves after six months was 41% lower than that of the plants at the Amami research site. The laboratory-grown plants appeared weak, and the stems turned and bent downward, as shown in Figure 11d. The plants grown in native soil looked healthiest among the three soils.

**Figure 12:**
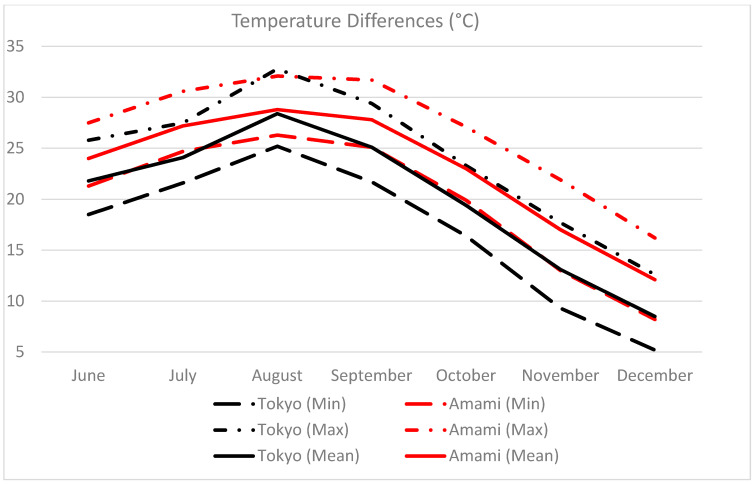
Comparison of average minimum, mean, and maximum temperatures between Tokyo and Amami (Kagoshima Prefecture)—source: Japan Meteorological Agency.

The germination of seedlings began with the development of the stem with a pair of leaves in the first month in all soil media, as shown in Figure 11b. After the third month, the average height of plants in all soil media was 28 cm above the soil surface, with an average number of leaves of 3.5. The average height and number of leaves grown in mangrove plants in all types of soil medium at the end of the sixth month were 38.3 cm and 4.3, respectively. The average increase in plant height and number of leaves in all soil media in the first, third, and sixth months were 3.7, 7.7, and 9.7 cm and 2, 1.5, and 0.8, respectively. With the arrival of new leaves, the plants lost their older leaves, which turned yellow and withered before dropping off see Figure 11c,d. Loss of leaves and decrease in leaf growth indicate lower leaf health because of environmental stresses [43]. This is probably because of the low temperature in November and December, as shown in Figure 12.

The air temperature differences in Tokyo and Amami for the period of six months from June to December 2019 are shown in Figure 12. On average, the temperature in Amami was 2.8 °C higher than in Tokyo. However, the maximum temperature in Tokyo (28.8 °C) was 0.4 °C higher than that in Amami (28.4 °C) in August. In December, the average temperature dropped to 8.5 °C in Tokyo, while that in Amami was 12.1 °C. The cold weather during the early winter season probably retarded the growth of mangrove plants and resulted in the withering of the leaves.

The diameter of the laboratory mangroves remained almost unchanged and was less than 4 mm on average, while the average stem diameter of Amami mangroves was 5.4 mm, which is 35% thicker than that of the laboratory mangroves. The Amami mangrove stems appeared tougher and had a deeper green color than those in the laboratory. The leaf surface of the Amami plants was also slightly thicker, whereas that of the laboratory mangroves was thinner and lighter in color.

The observations of the plants grown in the laboratory suggest that mangrove plants initially grown under a controlled environment do not necessarily show similar growth to that of the plants grown in the field. Laboratory-grown plants were thin, with reduced growth rate and leaf numbers of 20.8% and 41%, respectively, compared with the Amami mangroves, clearly suggesting that the laboratory mangroves were weaker. Seedlings raised in pots are often planted on the coast for mangrove restoration [31,49,50]. Such plants grown in a different location may not acclimatize themselves well and may not easily survive when transported and replanted in the field. In Sungai Haji Dorani, Malaysia, only 30% of the transplanted plants survived [51]. Given all these observations, it is ideal to replant mangroves directly in the required location rather than in nursery plantations. It is expected that portable reefs placed in front of the mangroves can safeguard seeds and young plants from hydrodynamic disturbances and promote the initial growth of mangroves.

## 4. Case Study: Design of Portable Reef as a Community-Based Breakwater

A portable reef was investigated. It was a community-based breakwater composed of a low-crested rubble mound with single-sized stones. In this section, a portable reef designed as a case study based on the wave and topographic conditions in Amami is described. Because many researchers have studied the stability, wave attenuation, and wave transmission of rubble breakwaters for several decades [52,53,54,55,56,57], the existing formulae were used to address the extent to which the portable reef can be reduced in size while maintaining favorable wave conditions for the growth of young mangroves.

### 4.1. Stability of Rubble Mound

A simple design is desirable to achieve an economical and community-oriented countermeasure. The design of extreme wave conditions would result in heavy stone weights in achieving sufficient structural stability, but such materials cannot be easily handled as community-based activities without the use of heavy equipment. Hence, extreme events, such as tropical cyclones, should be omitted from design considerations. Based on field observations and astronomical tide levels at the study site in Amami, 40 cm is considered the maximum water depth *d*. The breaking wave criteria proposed by Weggel (*H_B_* < 0.78*d*) lead to a maximum wave height of approximately 0.3 m for this depth condition. Hence, 0.3 m was the design wave height in this case analysis (Table 1). The wave period was assumed to vary from 2 to 3.5 s as a short wave in very shallow waters. Although the wave period seems negligible, it has a substantial impact on the portable reef and mangrove plants. Following the convention in coastal engineering, a significant wave (average of the upper one-third) was used as the design wave. In this case study, a rubble mound was designed that can withstand the design waves and confirm how well the transmitted waves can be mitigated.

The traditional method of designing rubble breakwaters assumes a stable structure with no damage or statically less than 5% damage levels [57]. A Hudson stability formula was developed from experimental investigations on a permeable breakwater subjected to nonovertopping waves. The equation states the relationship between the armor unit weight and the wave height at the toe of the structure, as shown in Equation (1) [58]:

(1) $$W=\;\frac{\gamma_{s}Hs^{3}}{K_{D}{\left(s-1\right)}^{3}cot\alpha }$$

where *W* is the weight of a single armor unit, *γ_s_* is the specific stone weight, *K_D_* is the dimensionless stability coefficient, *s* is the specific gravity of the armor unit, *α* is the structural slope angle, and *H_s_* is the significant wave height. Equation (1) does not consider the damage level, irregular wave conditions, wave period, storm duration, and permeability of stones.

**Figure 13:**
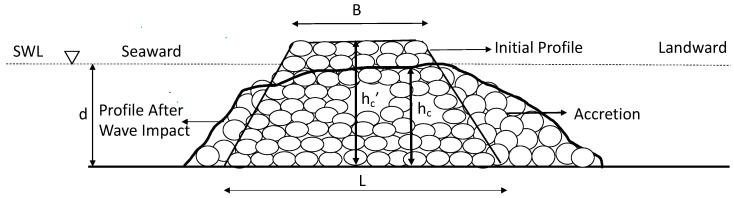
Typical reef profile before and after damage, adapted from Ahrens (1989) [58].

However, it is essential to allow deformation of the system to some extent, particularly in the case of smaller stones. Hence, the formula obtained by Ahrens [59], which designs a low-crested rubble-mound, reef-type breakwater without a multilayer cross section, was applied. Figure 13 illustrates the concept of the low-crested breakwater. Here, *h_c_*′ is the initial crest height, and *h_c_* is the crest height at the end of the wave impacts, based on an empirical equation from the experiment. In addition, B is the crest width (three median stones wide: 3D_n50_). The stability can be examined by considering the crest height that sunk as a result of continuous wave impacts.

**Figure 14:**
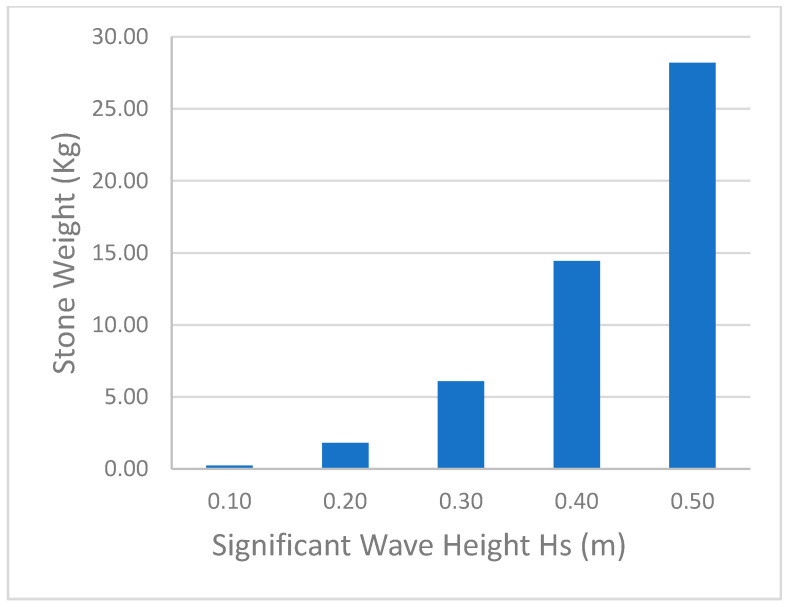
Required weight of single stone against a given wave height.

Equation (1) is analyzed for the range of significant wave heights (*H_s_*), with *K_D_* being 1.2 for quarry stone, smoothly rounded for breaking waves [57]. Here, $\gamma_{s}$ is taken as 2800 kg/m^3^, and the slope of the structure is considered to be 1V:2.5H, leading to a slope angle α of 21.6°. The relationship between *H_s_* and the armor unit weight is plotted in Figure 14. When 0.3 m is used as the design wave height, the lowest stone weight is calculated to be approximately 6 kg.

The structural stability with selected stone weights can be further investigated using Equations (2)–(4). The stability number (*N_s_*) of the unit is defined as [58]:

(2) $$N_{s}=\frac{H_{s}{}^{2/3}L^{1/3}}{\Delta D_{n50}}$$

where *L* is the wavelength calculated using the wave period (T) and water depth. The reduction in the crest height of the structure was estimated by the Equation (3) which was modified by Van der Meer after reanalyzing the data of Ahrens [58]:

(3) $$h_{c}=\sqrt{\frac{A_{t}}{a\exp N_{s}}}$$

(4) $$a=\;-0.028+0.045\;C^{\prime }+0.034\frac{{h_{c}}^{\prime }}{h}-6\times 10^{-9}B_{n}{}^{2}$$

**Figure 15:**
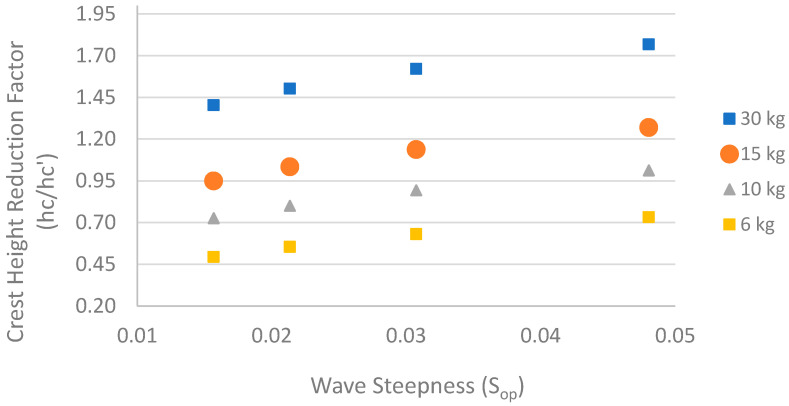
Crest height reduction factor (*h_c_/h_c_*′) versus wave steepness (*S_op_*).

Here, *A_t_* is the structural cross-sectional area (*Bh_c_*′+*C*′*h_c_*′^2^), C′ is the average structural slope, *N_s_* is the spectral stability number, B_n_ is the bulk number, and d is still water depth. Short wave periods in the range between T = 2 and 3.5 s are assumed to calculate the wavelength *L* = $\sqrt{gd}$T and wave steepness *S_op_* = 2π*H_s_*/gT^2^. The deformation in crest height (*h_c_*) can be estimated using Equations (3) and (4). Figure 15 shows a graph of the crest height reduction factor (*h_c_/h_c_*′) versus wave steepness. If *h_c_/h_c_*′ exceeds 1, the structure is fully stable, whereas, when *h_c_/h_c_*′ drops below 1, the structure is less stable [57]. The structural stability increases with the increase in wave steepness. A stone weight more than 15 kg showed *h_c_/h_c_*′ > 1, indicating a stable condition for the design wave. Hence, for the design considerations of stone, a weight of 15 kg can be used as an optimal weight, rather than 6 kg.

### 4.2. Wave Transmission and Cross-Sectional Design

Wave breaking and energy dissipation are promoted when waves are transmitted over a reef [60]. The degree of wave transmission is estimated by the coefficient of wave transmission (K_t_), which is defined by the transmitted wave divided by the incident wave heights (*H_t_/H_i_*). Wave transmission depends on the geometry of the reef, mainly on crest width and water depth, wave conditions, permeability, freeboard (crest height above water level) (*R_c_*), and wave steepness (*S_op_*) [61]. The prediction of the wave transmission characteristics of breakwaters has been studied, and equations for *K_t_* have been established. The following equation is used for the present analysis of wave transmission over a portable reef [57]:

(5) $$K_{t}=\left(0.031\;\frac{H_{i}}{D_{n50}}-0.24\right)\;\frac{R_{c}}{D_{n50}}-2.6\;S_{op}-0.05\frac{H_{i}}{D_{n50}}+0.85$$

**Figure 16:**
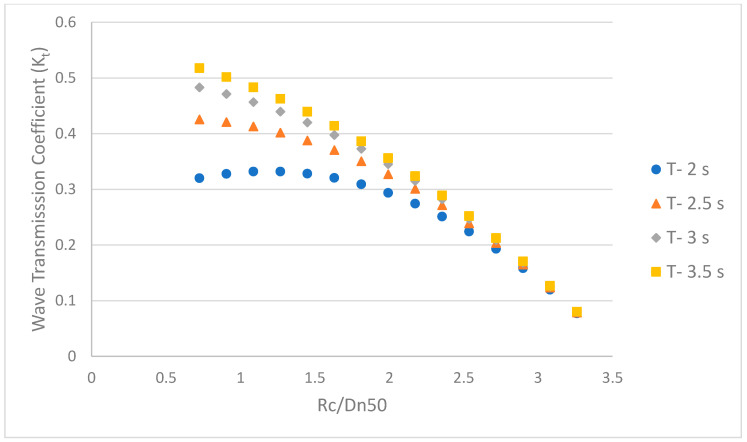
Wave transmission versus relative crest height.

Here, *K_t_* is derived for the proposed portable reef design (Figure 16. The *K_t_* value markedly varies with the change in the relative crest height (*R_c_/D_n_*_50_). The minimum *K_t_* and maximum *K_t_* expected of a portable reef breakwater fall between 0.07 and 0.51 for wave periods of 2 to 3.5 s. The lower the relative crest height, the higher the transmission. The lower the height of the breakwater, the smaller the stone volume required. However, a reduction in the freeboard increases the transmission of the waves and adversely affects the growth of mangroves. In the field experiments in Amami, it was found that mangroves of approximately one- or two-month-old plants were washed away by waves, even at a wave height of approximately 10 cm. Hence, *K_t_* needs to be set below 0.3 in the case of a wave height of 30 cm at the portable reef.

**Figure 17:**
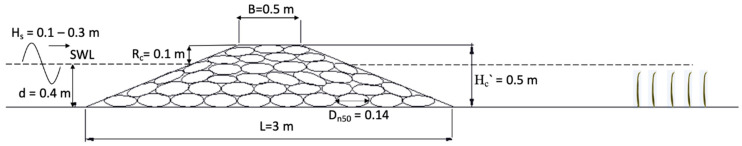
Example of a portable reef and mangrove planting.

As a result of these considerations, the breakwater cross section shown in Figure 17 was selected as one of the candidates to form the entire reef system as simply and feasibly as possible for the local community. The total number of stones needed to form a trapezoid per cubic meter was estimated as 145 (average of 15 kg each). Because of the low weight of the stone, construction can be accomplished without the use of heavy machinery if several workers collaborate. This is an advantage in areas where the ground is loose, such as where mangroves grow. There is a possibility that the reef top may sink a little bit owing to settlement, but it will be easy to replenish. A mangrove plantation is implemented after the reef is installed. However, plants may be washed away because of the disturbance caused by wave overtopping if planted immediately behind the reef. Therefore, it is recommended to maintain a certain distance between the reef and the plantation. It is necessary in future research to investigate how much distance is needed.

## 5. Conclusions

The restoration of mangrove forests has not always been successful, despite enormous attempts. In this study, the importance of protecting young mangroves from hydrodynamic disturbances was addressed to improve the implementation of plantations. Early growth of mangroves was studied both in the natural environment (a mangrove forest on Amami) and in a controlled environment (a laboratory in Tokyo). It was observed that plants could grow in any type of soil, even in the indoor environment, but the plants grown in the field looked healthier and stronger than those in the laboratory after six months. The mangrove growth test suggests that direct planting of seedlings in the restoration site is preferable rather than transporting germinated seedlings on a pot. Thus, a portable reef system was proposed to act as an effective wave attenuator to facilitate the growth of young mangrove seedlings. The Amami site was investigated to derive the design wave conditions necessary for protecting early mangroves. Sixth-month old mangrove plants can survive under normal wave conditions; hence, the service period of the portable reef system can be set as short as six months. The examination of the low-crested breakwater proposed by Ahrens was applied for the design of a portable reef system considering the structural stability and wave transmission ratio. As an example, the reef dimensions were designed and it was suggested that a 50-cm-high reef with approximately 15-kg stones is sufficient to protect against waves and effectively promote early mangrove growth. Once the mangroves have grown for about six months, the portable reef is no longer needed and can be dismantled and transported to other locations in the vicinity for reuse. In this way, community-based mangrove plantations should be able to continue in the long term without a high cost.

## Figures and Tables

**Table 1:** Design conditions.

Variables	Notation	Value
Wave heights	H	0.3 m
Wave periods	T	2 to 3.5 s
Water depth	d	0.4 m

## Data Availability

Not applicable.
